# DISASTER PREPAREDNESS OF COMMUNITY-DWELLING OLDER ADULTS

**DOI:** 10.1093/geroni/igad104.0689

**Published:** 2023-12-21

**Authors:** Lindsay Peterson

**Affiliations:** University of South Florida, Tampa, Florida, United States

## Abstract

Older adults who depend on others for care are especially vulnerable to harm in a disaster. However, the extent to which they are protected depends on the setting in which they live. State and federal regulations require nursing homes and assisted living communities to have preparedness plans that specifically address the needs of their residents. For older adults living at home, however, protections are far less prescriptive, and in some cases lacking altogether, despite the increasing numbers of disabled older adults receiving care in their homes. In this policy-based study, we examined disaster preparedness for community-dwelling older adults in high-disaster states with high populations of older adults. We found that while many of the states required home-based providers to respond to the post-disaster needs of their older adult clients, few took steps to ensure that before the storm they were prepared. In this study, the state of Florida was an exception, with policies calling for service providers to identify and contact at-risk clients before and after an event. The plan further identifies disaster preparedness as a key component of a livable community. Hurricane Ian struck Fort Myers, Florida, in September 2022. Older adults made up a majority of the 148 killed during and immediately after the storm. Many others lost their homes. In the context of this recent event, we will discuss not only the state and local policies, but the practices that affect the health and well-being of community-dwelling older adults faced with a major disaster.

